# Biomarkers for PVR in rhegmatogenous retinal detachment

**DOI:** 10.1371/journal.pone.0214674

**Published:** 2019-04-03

**Authors:** Souska Zandi, Isabel B. Pfister, Peter G. Traine, Christoph Tappeiner, Alain Despont, Robert Rieben, Magdalena Skowronska, Justus G. Garweg

**Affiliations:** 1 Swiss Eye Institute and Clinic for Vitreoretinal Diseases, Berner Augenklinik am Lindenhof-Spital, Bern, Switzerland; 2 Department of Ophthalmology, Inselspital, Bern University Hospital, University of Bern, Bern, Switzerland; 3 Department for Biomedical Research, University of Bern, Bern, Switzerland; 4 University of Bern, Bern, Switzerland; Massachusetts Eye & Ear Infirmary, Harvard Medical School, UNITED STATES

## Abstract

**Purpose:**

Various profibrotic and proinflammatory cytokines have been found upregulated in uncomplicated primary retinal detachment (pRD), but without providing a uniform picture. Here, we compare the cyto- and chemokine profiles in pRD with and without proliferative vitreoretinopathy (PVR) in an attempt to unravel relevant differences not in single cytokines, but in the cytokine profiles at diagnosis.

**Methods:**

Undiluted vitreous fluid (VF) was obtained at the beginning of surgery from 174 eyes with pRD without relevant PVR (maximally grade B; group 1; n = 81) and with moderate or advanced PVR requiring a gas tamponade (group 2; n = 49) or silicon oil filling (group 3; n = 44). VF of eyes undergoing macular hole (MH) surgery served as controls (group 4; *n* = 26). Forty-three cytokines were quantified in parallel using a multiplex cytokine analysis system (Bioplex). For all comparisons we applied Holm’s correction to control for multiple comparisons.

**Results:**

44.9% of group 2 eyes presented grade C1 and 55.1% C2-C3, whereas 86.4% of group 3 eyes exhibited a PVR grade of C2-D.

CCL19 was the only cytokine that displayed higher concentrations in the vitreous of eyes with PVR C1 compared to lower PVR grades. Eyes with PVR C2-D showed higher levels of CCL27, CXCL6, IL4, IL16, CXCL10, CCL8, CCL22, MIG/CXCL9, CCL15, CCL19, CCL 23 and CXCL12 compared to controls. Interestingly, no difference of cytokine levels was detected between C1 and C2-D PVR.

**Conclusions:**

CCL19 may represent a potential biomarker for early PVR progression that holds promise for future diagnostic and therapeutic applications.

## Introduction

Cell-signaling mediators, such as cytokines and chemokines are involved in the regulation of inflammatory processes, wound healing and scar formation [1]. In eyes with retinal detachment, elevated levels of a variety of cytokines and growth factors in the vitreous have been reported [2–7].

Retinal detachment (RD) induces cell migration and proliferation as well as the production of extracellular matrix proteins, which in turn lead to the development and contraction of vitreal and periretinal membranes, both hallmarks of proliferative vitreoretinopathy (PVR) [8,9,10]. PVR occurs in up to 10% of rhegmatogenous retinal detachment cases and is the major cause of poor functional outcomes after primarily successful RD surgery [11,12]. Depending on the duration and extent of RD, the accumulation of fibroblasts, collagen, and extracellular matrix components may co-occur with the formation of membranes on the vitreous and the interfaces of the entire retina, including the still attached parts. Previous studies found associations between PVR and significantly increased concentrations of certain pro-inflammatory cytokines and growth factors in the vitreous [13–19]. The majority of which did not exhibit any differences in their clinical relevance, except for IL-1, IL-6, IL-8, IL-10, TNF-alpha, IL1-beta, IFN gamma, ICAM-1, PDGF, MIF (macrophage inhibitory factor) and the chemokine ligands CCL2, CCL11, CCL17, CCL18, CCL19, CCL22, CXCL8, CXCL9 and CXCL10 [17,18,20].

The aim of this study was to not only focus on single cytokines in the vitreous fluid (VF) when comparing between cases with and without relevant PVR, but to provide a much broader comparison of profiles of pro-inflammatory and pro-fibrotic cytokines in the vitreous fluid (VF) which should facilitate an improved differentiation of their relative importance with respect to the pathophysiological process of PVR.

## Patients and methods

### Patients

The investigation was designed as a prospective study involving consecutive patients undergoing pars plana vitrectomy for the treatment of primary retinal detachment with and without PVR and subsequent gas or silicon oil filling. All surgeries had been performed by the same surgeon at the Berner Augenklinik am Lindenhofspital, Bern, Switzerland. Patients with systemic or ocular comorbidities that may potentially influence ocular cytokine levels were excluded, i.e. patients with diabetes mellitus, known rheumatic and autoimmune diseases, systemic treatments involving corticosteroids or immunomodulatory drugs, vitreous hemorrhaging, uveitis, glaucoma, or any concomitant retinal pathology, or who had undergone intraocular surgery or treatment within six months of the RD diagnosis. If both eyes were affected, only the first operated eye was included. The exclusion criteria apply also to the control group.

The study was approved by the Ethics committee of the University of Bern (KEK no. 152/08), and is fully compliant with the tenets of the Declaration of Helsinki. Each participant provided their informed written consent to the use of their biological materials and clinical data.

### PVR grading and patient grouping

**Fig 1:**
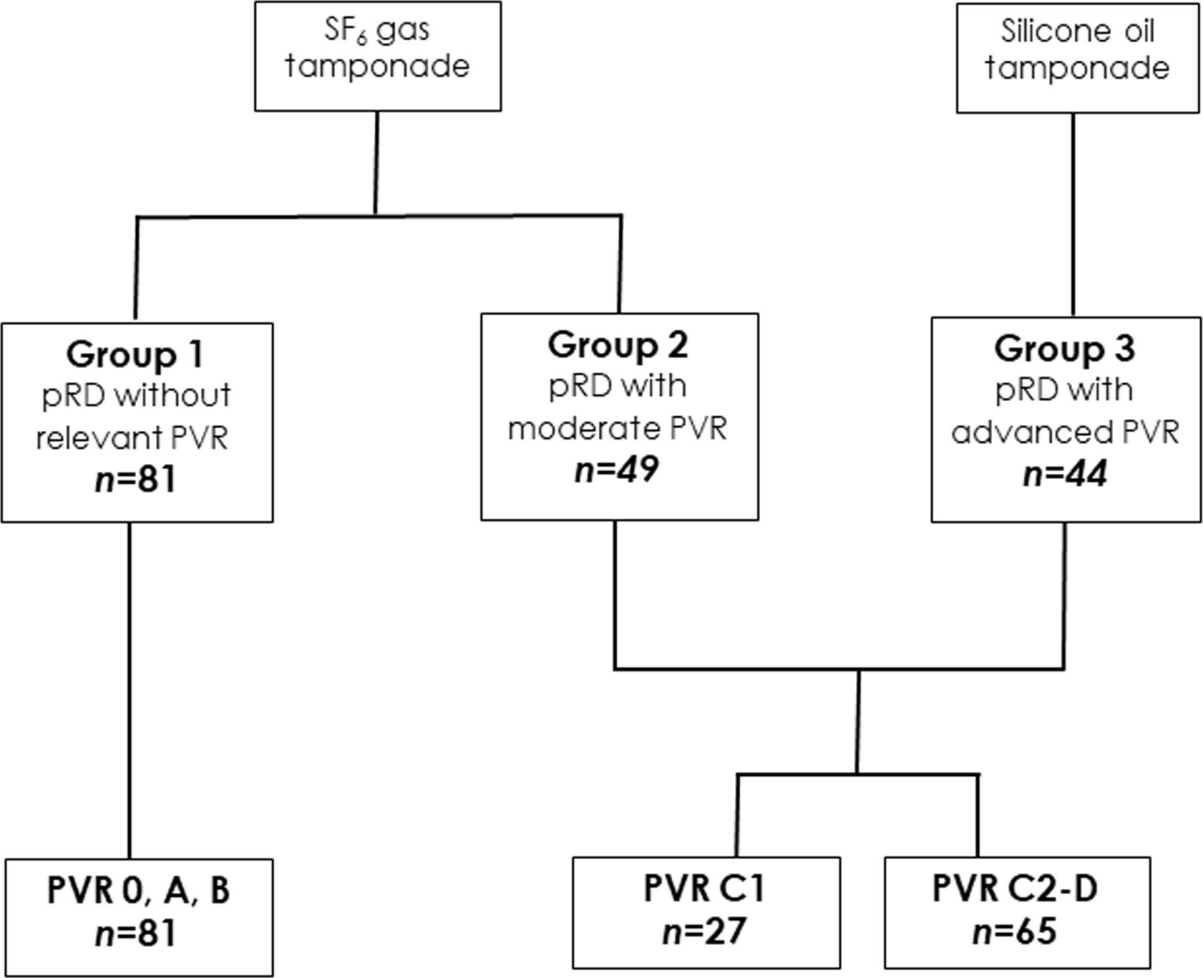
Schematic diagram of groups and PVR grades. Group 1 eyes showed a maximal PVR grade A or B (no relevant vascular wrinkling), whereas 44.9% of group 2 presented a PVR grade C1 and in 55.1% grades C2 or C3. However, 86.4% of eyes in group 3 exhibited a PVR grade of C2-D and required silicone oil tamponade. Therefore, we labeled these groups as “without relevant PVR” (group 1), moderate (group 2) and advanced PVR (group 3).

Following recommendations from the “Retina Society Terminology Committee (1983)” [21], we classified patients based on PVR severity into four stages: A (minimal), B, C, and D (massive). For the purpose of this study, we considered the risk of developing postoperative PVR to be similar in pRD patients without PVR and pRD patients with low PVR severity (grades A or B) (denoted collectively as group 1, *n* = 81). On the basis of this classification system, PVR grade C was further subcategorized into C1 –C3, with the numerals 1–3 referring to the number of quadrants with visible PVR membrane formation. If all 4 quadrants were affected, the severity was defined as grade D. Since accurate grading in advanced PVR may be difficult, wide field images (Optos TX200, Optos Inc, Dunfermline, Scotland) were obtained from all eyes prior to surgery. To examine postoperative PVR risk, patients with advanced PVR were grouped according to the intraoperative decision regarding the type of tamponade (Fig 1): eyes receiving an SF_6_ gas tamponade were categorized as group 2 (*n* = 49, those in need of a silicone oil tamponade are group 3 (*n* = 44), while those undergoing macular hole (MH) surgery with SF_6_ gas tamponade served as control (group 4; *n* = 26).

### Handling of vitreous fluid samples

Approximately 500 microliters of undiluted VF were collected at the beginning of pars plana vitrectomy. After harvesting, the VF initially stored at -20°C and moved to -80°C after maximally 2 months where it remained until the final analysis which was performed on all samples simultaneously.

### Cytokine analyses

The samples were analyzed using a multiplex system (Bio-Plex 100 array reader with Bio-Plex Manager software version 6.1; Bio-Rad, Hercules, CA, USA). With this highly sensitive technique, multiple analytes can be detected in parallel using a single small volume sample. We quantified the concentrations of 43 cytokines in each vitreous sample (Tables 1–6). All analytic procedures were performed following the manufacturer’s instructions. In short, magnetic microspheres, tagged with a fluorescent label were coupled to specific capture antibodies and mixed with samples containing unknown cytokine quantities before introducing biotinylated detection antibodies and Streptavidin R-Phycoerythrin. The mixture was then analyzed by flow cytometry. The instrument’s two lasers identify the microsphere type and quantify the amount of bound antigen. On each test plate we ran a duplicate concentration standard in parallel for each cytokine. The measurements were performed in a blinded manner by a laboratory technician who was experienced in the execution of this technique.

**Table 1:** Mean concentrations (pg/ml) and standard deviations (SD) of cytokines in the vitreous of eyes with primary retinal detachment without PVR (pRD) and in eyes with primary retinal detachment with moderate PVR treated with SF_6_ gas tamponade.

Cytokine	pRD		moderate PVR		Mann-Whitney U test
	Mean (pg/ml)	SD	Mean (pg/ml)	SD	
**CCL21**	2381.8	5022.1	1994.3	2675.1	*p* = 0.333
**CXCL13**	1.9	2.8	2.3	2.7	*p* = 0.01795
**CCL27**	10.6	41.0	11.5	24.2	*p* = 0.08551
**CXCL5**	164.7	195.1	189.8	184.3	*p* = 0.2683
**CCL11**	13.1	16.1	16.4	17.2	*p* = 0.04332
**CCL24**	21.4	22.2	22.1	14.0	*p* = 0.1448
**CCL26**	9.9	14.5	12.1	15.5	*p* = 0.03833
**CX3CL1**	67.7	70.9	88.9	91.0	*p* = 0.05434
**CXCL6**	2.3	3.8	3.5	4.8	*p* = 0.08683
**GM-CSF**	45.9	18.0	49.5	21.2	*p* = 0.3153
**CXCL1**	66.4	64.0	80.3	62.0	*p* = 0.03006
**CXCL2**	22.5	51.1	46.6	96.9	*p* = 0.01459
**CCL1**	35.5	59.3	49.0	68.1	*p* = 0.02713
**IFN-γ**	8.7	11.9	11.8	14.3	*p* = 0.1089
**IL-1β**	1.5	2.2	2.3	3.1	*p* = 0.02623
**IL-2**	1.5	1.8	1.8	1.3	*p* = 0.02031
**IL-4**	3.0	4.8	4.8	5.7	*p* = 0.03141
**IL-6**	136.0	349.1	418.3	1393.3	*p* = 0.0312
**IL-8/CXCL8**	36.5	51.2	46.1	51.9	*p* = 0.04618
**IL-10**	7.2	5.4	8.6	4.6	*p* = 0.0295
**IL-16**	57.6	45.1	92.3	110.0	*p* = 0.01556
**CXCL10**	350.3	1529.3	303.0	628.0	*p* = 0.07788
**CXCL11**	5.0	8.1	5.7	7.2	*p* = 0.1627
**CCL2**	1602.8	1597.1	1892.5	1415.4	*p* = 0.04438
**CCL8**	12.4	30.6	13.2	18.7	*p* = 0.03119
**CCL7**	21.8	26.0	29.3	32.4	*p* = 0.08307
**CCL13**	2.3	2.4	3.1	3.3	*p* = 0.01818
**CCL22**	14.0	17.3	15.1	11.6	*p* = 0.2365
**MIF**	101278.2	99480.3	113452.4	105774.0	*p* = 0.5058
**MIG/CXCL9**	304.1	2254.6	138.7	666.4	*p* = 0.09597
**CCL3**	3.1	2.7	4.0	3.2	*p* = 0.02018
**CCL15**	712.1	600.2	930.3	1049.4	*p* = 0.3806
**CCL20**	10.9	14.9	17.4	25.6	*p* = 0.0232
**CCL19**	38.1	62.8	47.4	48.9	*p* = 0.002001
**CCL23**	17.4	19.9	21.6	24.6	*p* = 0.1465
**CXCL16**	812.5	313.6	911.5	312.8	*p* = 0.0816
**CXCL12**	169.6	233.9	194.8	137.4	*p* = 0.003913
**CCL17**	6.4	19.2	8.4	15.1	*p* = 0.0775
**CCL25**	384.9	498.9	464.4	568.1	*p* = 0.08286
**TNF-α**	13.3	17.8	15.9	13.8	*p* = 0.04564
**TGF-β1**	100.1	215.5	34.5	74.2	*p* = 0.08435
**TGF-β2**	1243.6	823.9	871.0	673.7	*p* = 0.01038
**TGF-β3**	12.3	26.4	6.1	12.6	*p* = 0.2018

**Table 2:** Mean concentrations (pg/ml) and standard deviations (SD) of cytokines in the vitreous of eyes with primary retinal detachment without PVR (pRD) and in eyes with primary retinal detachment with advanced PVR requiring silicone oil tamponade.

Cytokine	pRD		advanced PVR		Mann-Whitney U test
	Mean (pg/ml)	SD	Mean (pg/ml)	SD	
**CCL21**	2381.8	5022.1	4226.2	7403.1	*p* = 0.003323
**CXCL13**	1.9	2.8	4.1	8.0	*p* = 0.001729
**CCL27**	10.6	41.0	37.3	102.3	***p* = 1.27E-06**
**CXCL5**	164.7	195.1	178.7	195.4	*p* = 0.7854
**CCL11**	13.1	16.1	20.8	22.2	*p* = 0.007636
**CCL24**	21.4	22.2	37.1	45.8	*p* = 0.00504
**CCL26**	9.9	14.5	17.3	19.2	**p = 0.001253**
**CX3CL1**	67.7	70.9	98.2	92.3	*p* = 0.009529
**CXCL6**	2.3	3.8	6.9	8.5	***p* = 0.0002024**
**GM-CSF**	45.9	18.0	52.8	19.2	*p* = 0.04683
**CXCL1**	66.4	64.0	83.7	59.9	*p* = 0.01489
**CXCL2**	22.5	51.1	71.1	112.7	*p* = 0.004821
**CCL1**	35.5	59.3	65.6	97.6	*p* = 0.02381
**IFN-γ**	8.7	11.9	17.2	18.2	*p* = 0.00204
**IL-1β**	1.5	2.2	2.3	3.5	*p* = 0.08084
**IL-2**	1.5	1.8	2.0	1.5	*p* = 0.01801
**IL-4**	3.0	4.8	6.5	6.6	***p* = 0.0002875**
**IL-6**	136.0	349.1	1348.3	6939.1	*p* = 0.007979
**IL-8/CXCL8**	36.5	51.2	55.2	59.7	*p* = 0.007763
**IL-10**	7.2	5.4	10.0	6.1	*p* = 0.006387
**IL-16**	57.6	45.1	119.4	113.4	***p* = 0.0002374**
**CXCL10**	350.3	1529.3	418.4	590.2	***p* = 3.39E-05**
**CXCL11**	5.0	8.1	7.9	10.0	*p* = 0.03648
**CCL2**	1602.8	1597.1	1815.8	1941.9	*p* = 0.7604
**CCL8**	12.4	30.6	19.6	24.8	***p* = 0.0003082**
**CCL7**	21.8	26.0	35.9	35.3	*p* = 0.01288
**CCL13**	2.3	2.4	3.9	3.8	*p* = 0.01031
**CCL22**	14.0	17.3	27.8	25.4	***p* = 0.0001539**
**MIF**	101278.2	99480.3	138533.6	128551.1	*p* = 0.07621
**MIG/CXCL9**	304.1	2254.6	470.3	1452.7	***p* = 1.05E-05**
**CCL3**	3.1	2.7	4.6	4.3	*p* = 0.007524
**CCL15**	712.1	600.2	1901.6	2228.4	***p* = 0.0001252**
**CCL20**	10.9	14.9	20.3	35.9	*p* = 0.00208
**CCL19**	38.1	62.8	93.7	143.2	***p* = 4.26E-05**
**CCL23**	17.4	19.9	37.9	44.1	***p* = 1.27E-05**
**CXCL16**	812.5	313.6	996.9	356.6	*p* = 0.003985
**CXCL12**	169.6	233.9	330.4	335.7	***p* = 2.07E-05**
**CCL17**	6.4	19.2	14.0	26.8	*p* = 0.006175
**CCL25**	384.9	498.9	613.3	628.8	*p* = 0.005957
**TNF-α**	13.3	17.8	19.1	15.7	*p* = 0.005545
**TGF-β1**	100.1	215.5	118.6	214.8	*p* = 0.5693
**TGF-β2**	1243.6	823.9	1221.0	1213.8	*p* = 0.2869
**TGF-β3**	12.3	26.4	15.0	24.7	*p* = 0.7028

**Table 3:** Mean concentrations (pg/ml) and standard deviations (SD) of cytokines in the vitreous of eyes with primary retinal detachment with moderate PVR treated with SF_6_ gas tamponade and in eyes with primary retinal detachment with advanced PVR requiring silicone oil tamponade.

Cytokine	moderate PVR		advanced PVR		Mann-Whitney U test
	Mean (pg/ml)	SD	Mean (pg/ml)	SD	
**CCL21**	1994.3	2675.1	4226.2	7403.1	*p* = 0.07548
**CXCL13**	2.3	2.7	4.1	8.0	*p* = 0.1684
**CCL27**	11.5	24.2	37.3	102.3	*p* = 0.003019
**CXCL5**	189.8	184.3	178.7	195.4	*p* = 0.5609
**CCL11**	16.4	17.2	20.8	22.2	*p* = 0.453
**CCL24**	22.1	14.0	37.1	45.8	*p* = 0.166
**CCL26**	12.1	15.5	17.3	19.2	*p* = 0.2041
**CX3CL1**	88.9	91.0	98.2	92.3	*p* = 0.4059
**CXCL6**	3.5	4.8	6.9	8.5	*p* = 0.03418
**GM-CSF**	49.5	21.2	52.8	19.2	*p* = 0.5081
**CXCL1**	80.3	62.0	83.7	59.9	*p* = 0.6035
**CXCL2**	46.6	96.9	71.1	112.7	*p* = 0.6324
**CCL1**	49.0	68.1	65.6	97.6	*p* = 0.8024
**IFN-γ**	11.8	14.3	17.2	18.2	*p* = 0.1102
**IL-1β**	2.3	3.1	2.3	3.5	*p* = 0.7817
**IL-2**	1.8	1.3	2.0	1.5	*p* = 0.7002
**IL-4**	4.8	5.7	6.5	6.6	*p* = 0.1649
**IL-6**	418.3	1393.3	1348.3	6939.1	*p* = 0.4236
**IL-8/CXCL8**	46.1	51.9	55.2	59.7	*p* = 0.4886
**IL-10**	8.6	4.6	10.0	6.1	*p* = 0.3951
**IL-16**	92.3	110.0	119.4	113.4	*p* = 0.1934
**CXCL10**	303.0	628.0	418.4	590.2	*p* = 0.01602
**CXCL11**	5.7	7.2	7.9	10.0	*p* = 0.4862
**CCL2**	1892.5	1415.4	1815.8	1941.9	*p* = 0.2015
**CCL8**	13.2	18.7	19.6	24.8	*p* = 0.1095
**CCL7**	29.3	32.4	35.9	35.3	*p* = 0.3634
**CCL13**	3.1	3.3	3.9	3.8	*p* = 0.4393
**CCL22**	15.1	11.6	27.8	25.4	*p* = 0.008345
**MIF**	113452.4	105774.0	138533.6	128551.1	*p* = 0.2813
**MIG/CXCL9**	138.7	666.4	470.3	1452.7	*p* = 0.002084
**CCL3**	4.0	3.2	4.6	4.3	*p* = 0.7033
**CCL15**	930.3	1049.4	1901.6	2228.4	*p* = 0.007758
**CCL20**	17.4	25.6	20.3	35.9	*p* = 0.3227
**CCL19**	47.4	48.9	93.7	143.2	*p* = 0.2001
**CCL23**	21.6	24.6	37.9	44.1	***p* = 0.0008617**
**CXCL16**	911.5	312.8	996.9	356.6	*p* = 0.1895
**CXCL12**	194.8	137.4	330.4	335.7	*p* = 0.05835
**CCL17**	8.4	15.1	14.0	26.8	*p* = 0.3305
**CCL25**	464.4	568.1	613.3	628.8	*p* = 0.2154
**TNF-α**	15.9	13.8	19.1	15.7	*p* = 0.338
**TGF-β1**	34.5	74.2	118.6	214.8	*p* = 0.0453
**TGF-β2**	871.0	673.7	1221.0	1213.8	*p* = 0.1988
**TGF-β3**	1994.3	2675.1	15.0	24.7	*p* = 0.1831

**Table 4:** Mean concentrations (pg/ml) and standard deviations (SD) of cytokines in the vitreous of eyes with primary retinal detachment with PVR grade B or less and in eyes with PVR C1.

Cytokine	PVR grade B or less		PVR grade C1		Mann-Whitney U test
	Mean (pg/ml)	SD	Mean (pg/ml)	SD	
**CCL21**	2381.8	5022.1	2617.5	3303.7	*p* = 0.141
**CXCL13**	1.9	2.8	2.2	2.2	*p* = 0.01794
**CCL27**	10.6	41.0	11.0	21.0	*p* = 0.1178
**CXCL5**	164.7	195.1	211.5	196.1	*p* = 0.1571
**CCL11**	13.1	16.1	20.1	20.0	*p* = 0.02337
**CCL24**	21.4	22.2	24.3	16.1	*p* = 0.1088
**CCL26**	9.9	14.5	15.5	19.3	*p* = 0.02906
**CX3CL1**	67.7	70.9	95.3	93.8	*p* = 0.05406
**CXCL6**	2.3	3.8	3.7	5.7	*p* = 0.1644
**GM-CSF**	45.9	18.0	51.1	20.6	*p* = 0.2547
**CXCL1**	66.4	64.0	84.9	63.9	*p* = 0.02091
**CXCL2**	22.5	51.1	52.9	119.0	*p* = 0.09327
**CCL1**	35.5	59.3	60.2	79.5	*p* = 0.02523
**IFN-γ**	8.7	11.9	12.6	15.2	*p* = 0.1052
**IL-1β**	1.5	2.2	1.8	1.8	*p* = 0.2038
**IL-2**	1.5	1.8	1.8	1.3	*p* = 0.05152
**IL-4**	3.0	4.8	5.1	6.2	*p* = 0.0411
**IL-6**	136.0	349.1	572.6	1834.9	*p* = 0.1652
**IL-8/CXCL8**	36.5	51.2	43.5	43.5	*p* = 0.09129
**IL-10**	7.2	5.4	9.4	4.6	*p* = 0.01694
**IL-16**	57.6	45.1	73.6	45.4	*p* = 0.07492
**CXCL10**	350.3	1529.3	387.8	803.8	*p* = 0.1709
**CXCL11**	5.0	8.1	7.1	8.6	*p* = 0.07255
**CCL2**	1602.8	1597.1	1951.5	1681.5	*p* = 0.0596
**CCL8**	12.4	30.6	14.9	23.1	*p* = 0.06931
**CCL7**	21.8	26.0	34.0	39.3	*p* = 0.1071
**CCL13**	2.3	2.4	3.6	3.8	*p* = 0.01105
**CCL22**	14.0	17.3	18.2	21.1	*p* = 0.3609
**MIF**	101278.2	99480.3	124323.2	117135.7	*p* = 0.4085
**MIG/CXCL9**	304.1	2254.6	222.7	895.7	*p* = 0.07377
**CCL3**	3.1	2.7	4.5	4.0	*p* = 0.03388
**CCL15**	712.1	600.2	1166.5	1704.2	*p* = 0.4289
**CCL20**	10.9	14.9	15.2	17.0	*p* = 0.05405
**CCL19**	38.1	62.8	55.8	55.5	***p* = 0.0009894**
**CCL23**	17.4	19.9	22.9	27.8	*p* = 0.1199
**CXCL16**	812.5	313.6	994.4	339.3	*p* = 0.02015
**CXCL12**	169.6	233.9	211.9	156.9	*p* = 0.006792
**CCL17**	6.4	19.2	10.4	18.5	*p* = 0.06664
**CCL25**	384.9	498.9	571.1	684.8	*p* = 0.03157
**TNF-α**	13.3	17.8	18.6	16.2	*p* = 0.01663
**TGF-β1**	100.1	215.5	41.8	134.9	*p* = 0.06634
**TGF-β2**	1243.6	823.9	899.5	677.8	*p* = 0.06155
**TGF-β3**	12.3	26.4	6.2	16.1	*p* = 0.08247

**Table 5:** Mean concentrations (pg/ml) and standard deviations (SD) of cytokines in the vitreous of eyes with primary retinal detachment with PVR grade B or less and in eyes with primary retinal detachment and PVR C2-D.

Cytokine	PVR grade B or less		PVR grade C2 or higher		Mann-Whitney U test
	Mean (pg/ml)	SD	Mean (pg/ml)	SD	
**CCL21**	2381.8	5022.1	3268.7	6271.7	*p* = 0.02321
**CXCL13**	1.9	2.8	3.6	6.9	*p* = 0.001814
**CCL27**	10.6	41.0	29.4	86.2	***p* = 1.75E-05**
**CXCL5**	164.7	195.1	174.5	187.2	*p* = 0.7461
**CCL11**	13.1	16.1	18.0	19.9	*p* = 0.01595
**CCL24**	21.4	22.2	31.6	38.8	*p* = 0.009679
**CCL26**	9.9	14.5	14.4	16.8	*p* = 0.002285
**CX3CL1**	67.7	70.9	93.3	91.4	*p* = 0.0134
**CXCL6**	2.3	3.8	5.7	7.4	***p* = 0.0004163**
**GM-CSF**	45.9	18.0	51.7	19.5	*p* = 0.06252
**CXCL1**	66.4	64.0	81.1	60.2	*p* = 0.01964
**CXCL2**	22.5	51.1	57.3	96.8	*p* = 0.002432
**CCL1**	35.5	59.3	56.0	85.9	*p* = 0.02527
**IFN-γ**	8.7	11.9	15.4	17.0	*p* = 0.004325
**IL-1β**	1.5	2.2	2.5	3.7	*p* = 0.01877
**IL-2**	1.5	1.8	1.9	1.5	*p* = 0.01031
**IL-4**	3.0	4.8	5.9	6.2	***p* = 0.0005886**
**IL-6**	136.0	349.1	986.2	5718.1	*p* = 0.003728
**IL-8/CXCL8**	36.5	51.2	52.1	59.8	*p* = 0.009737
**IL-10**	7.2	5.4	9.2	5.7	*p* = 0.01423
**IL-16**	57.6	45.1	118.1	128.7	***p* = 0.0002389**
**CXCL10**	350.3	1529.3	345.9	521.2	***p* = 0.0001969**
**CXCL11**	5.0	8.1	6.7	8.8	*p* = 0.0757
**CCL2**	1602.8	1597.1	1832.7	1693.6	*p* = 0.3683
**CCL8**	12.4	30.6	16.7	21.7	***p* = 0.0006981**
**CCL7**	21.8	26.0	32.0	31.8	*p* = 0.01758
**CCL13**	2.3	2.4	3.5	3.6	*p* = 0.01689
**CCL22**	14.0	17.3	22.4	20.2	***p* = 0.001134**
**MIF**	101278.2	99480.3	127494.3	118058.1	*p* = 0.1139
**MIG/CXCL9**	304.1	2254.6	330.3	1208.3	***p* = 0.0001447**
**CCL3**	3.1	2.7	4.2	3.7	*p* = 0.005434
**CCL15**	712.1	600.2	1502.8	1806.4	***p* = 0.0007773**
**CCL20**	10.9	14.9	20.3	35.1	*p* = 0.002397
**CCL19**	38.1	62.8	75.7	122.2	***p* = 0.0001397**
**CCL23**	17.4	19.9	32.3	38.8	***p* = 0.0002163**
**CXCL16**	812.5	313.6	945.8	324.0	*p* = 0.01275
**CXCL12**	169.6	233.9	280.8	291.1	***p* = 4.18E-05**
**CCL17**	6.4	19.2	11.2	22.9	*p* = 0.01808
**CCL25**	384.9	498.9	526.9	566.7	*p* = 0.0152
**TNF-α**	13.3	17.8	17.0	14.3	*p* = 0.01748
**TGF-β1**	100.1	215.5	88.9	171.9	*p* = 0.8986
**TGF-β2**	1243.6	823.9	1107.2	1078.3	*p* = 0.08566
**TGF-β3**	12.3	26.4	12.1	20.9	*p* = 0.781

**Table 6:** Mean concentrations (pg/ml) and standard deviations (SD) of cytokines in the vitreous of eyes with primary retinal detachment with PVR grade C1, and in eyes with PVR grade C2 or higher.

Cytokine	PVR C1		PVR C2 or higher		Mann-Whitney U test
	Mean (pg/ml)	SD	Mean (pg/ml)	SD	
**CCL21**	2617.5	3303.7	3268.7	6271.7	*p* = 0.8537
**CXCL13**	2.2	2.2	3.6	6.9	*p* = 0.6189
**CCL27**	11.0	21.0	29.4	86.2	*p* = 0.05675
**CXCL5**	211.5	196.1	174.5	187.2	*p* = 0.2931
**CCL11**	20.1	20.0	18.0	19.9	*p* = 0.665
**CCL24**	24.3	16.1	31.6	38.8	*p* = 0.797
**CCL26**	15.5	19.3	14.4	16.8	*p* = 0.9795
**CX3CL1**	95.3	93.8	93.3	91.4	*p* = 0.9897
**CXCL6**	3.7	5.7	5.7	7.4	*p* = 0.1623
**GM-CSF**	51.1	20.6	51.7	19.5	*p* = 0.8977
**CXCL1**	84.9	63.9	81.1	60.2	*p* = 0.625
**CXCL2**	52.9	119.0	57.3	96.8	*p* = 0.6923
**CCL1**	60.2	79.5	56.0	85.9	*p* = 0.6037
**IFN-γ**	12.6	15.2	15.4	17.0	*p* = 0.5475
**IL-1β**	1.8	1.8	2.5	3.7	*p* = 0.589
**IL-2**	1.8	1.3	1.9	1.5	*p* = 0.8705
**IL-4**	5.1	6.2	5.9	6.2	*p* = 0.5622
**IL-6**	572.6	1834.9	986.2	5718.1	*p* = 0.1674
**IL-8/CXCL8**	43.5	43.5	52.1	59.8	*p* = 0.7543
**IL-10**	9.4	4.6	9.2	5.7	*p* = 0.6099
**IL-16**	73.6	45.4	118.1	128.7	*p* = 0.181
**CXCL10**	387.8	803.8	345.9	521.2	*p* = 0.1597
**CXCL11**	7.1	8.6	6.7	8.8	*p* = 0.7028
**CCL2**	1951.5	1681.5	1832.7	1693.6	*p* = 0.3725
**CCL8**	14.9	23.1	16.7	21.7	*p* = 0.3611
**CCL7**	34.0	39.3	32.0	31.8	*p* = 0.8067
**CCL13**	3.6	3.8	3.5	3.6	*p* = 0.5598
**CCL22**	18.2	21.1	22.4	20.2	*p* = 0.153
**MIF**	124323.2	117135.7	127494.3	118058.1	*p* = 0.7543
**MIG/CXCL9**	222.7	895.7	330.3	1208.3	*p* = 0.2169
**CCL3**	4.5	4.0	4.2	3.7	*p* = 0.8807
**CCL15**	1166.5	1704.2	1502.8	1806.4	*p* = 0.081
**CCL20**	15.2	17.0	20.3	35.1	*p* = 0.6099
**CCL19**	55.8	55.5	75.7	122.2	*p* = 0.7739
**CCL23**	22.9	27.8	32.3	38.8	*p* = 0.1042
**CXCL16**	994.4	339.3	945.8	324.0	*p* = 0.6464
**CXCL12**	211.9	156.9	280.8	291.1	*p* = 0.474
**CCL17**	10.4	18.5	11.2	22.9	*p* = 0.9853
**CCL25**	571.1	684.8	526.9	566.7	*p* = 0.9044
**TNF-α**	18.6	16.2	17.0	14.3	*p* = 0.6099
**TGF-β1**	41.8	134.9	88.9	171.9	*p* = 0.04595
**TGF-β2**	899.5	677.8	1107.2	1078.3	*p* = 0.5229
**TGF-β3**	6.2	16.1	12.1	20.9	*p* = 0.05585

### Statistical analyses

By performing a Shapiro-Wilk test we found that our data were not normally distributed. As a result, we employed a non-parametric Mann-Whitney U test for the inter-group and intra-group comparisons. A p-value of <0.05 was considered to be significant. Since multiple comparisons increase the risk of introducing a Type-I error, we applied the sequentially rejective Bonferroni correction (Holm’s correction) to control for this type of error without introducing additional Type II errors [22,23]. This means that the p-value must be divided by the number of tests run in parallel, resulting in an adjusted level of statistical significance of p = 0.05/43 = 0.00116. All statistical analyses were performed using R (package FSA, software version 3.4.0).

## Results

### Patients

The eyes of 174 consecutive patients admitted to our clinic for primary retinal detachment met the inclusion criteria (group 1 n = 81; group 2 n = 49; group 3 n = 44). Thereof, 92 eyes were phakic and 82 eyes were pseudophakic (group1: *n* = 40, group 2: *n* = 17, group 3: *n* = 25). In the control group (group 4), 26 patients with macular hole underwent pars plana vitrectomy, all of which were phakic before surgery. Since we recently showed that the lens status does not significantly influence the cytokine profiles in the VF phakic and pseudophakic eyes in each group were pooled [24].

The mean age in group 4 was higher than in groups 1 and 2 while similar to group 3 (group 4 (control): 67.5 ± 8.2 years; group 1: 61.6 ± 14.3 years; group 2: 61.9± 9.6; group 3: 68.5± 14.1). While the control group (group 4) and group 2 were predominantly female (group 4: 19.2% males, 80.8% females, group 2: 38.8% males, 60.2% females) group 1 and 3 contained male majorities (group 1: 65.4% males; group 3: 59.1% males). However there were no significant differences in gender between the RD groups.

### Comparison of cytokine profiles in macular hole and pRD

**Fig 2:**
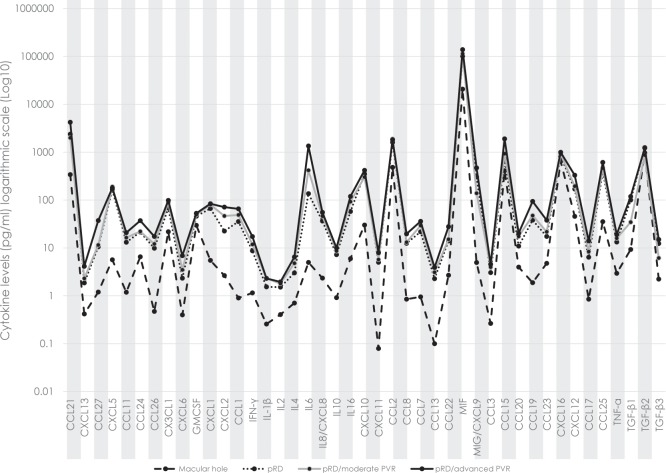
Cytokine levels in the vitreous fluid. Comparing cytokine levels in the vitreous (VF) of eyes of patients with primary retinal detachment without PVR (pRD; *n* = **81**) with moderate PVR (*n* = **49**), with advanced PVR (*n* = **44**), and eyes with macular holes.

Compared to the control group, pRD patients both with or without PVR displayed significantly higher levels of all chemo- and cytokines in the VF, except for TGF beta-1 and -2, even after correction for multiple comparisons (Fig 2).

Comparison of cytokine profiles in eyes with pRD without relevant PVR (group 1) and with PVR requiring a gas (group 2) or silicone oil tamponade (group 3)

We found no significant differences in the VF chemo- or cytokine levels between Groups 1 and 2 (Table 1).

In Group 3, however, the concentrations of 13 chemo-and cytokines (CCL27, CCL26, CXCL6, IL-4, IL-16, CXCL10, CCL8, CCL22, MIG/CXCL9, CCL15, CCL19, CCL23, CXCL12) were elevated compared to Group 1 (Table 2). We observed PVR grade of C2 or higher (C2 to D) in 86.4% of patients from Group 3.Comparison of cytokine profiles in eyes requiring gas (group 2) or silicone oil tamponade (group 3)A comparison between Groups 2 and 3 yielded only one cytokine, CCL23, with significantly elevated VF levels in group 3 (Table 3).

### Comparing cytokine profiles from eyes with pRD and PVR grade A or B to eyes with PVR grade C1

Patients with pRD with PVR grade B or less displayed one cytokine (CCL19) with elevated levels in comparison to patients with PVR C1, indicating an early change towards more severe PVR. When compared to patients with more severe PVR (grades C2 to D) the latter showed 12 cytokines with significantly higher VF levels: CCL27, CXCL6, IL4, IL16, CXCL10, CCL8, CCL22, MIG/CXCL9, CCL15, CCL19, CCL 23 and CXCL12 (Tables 4–6).

**Fig 3:**
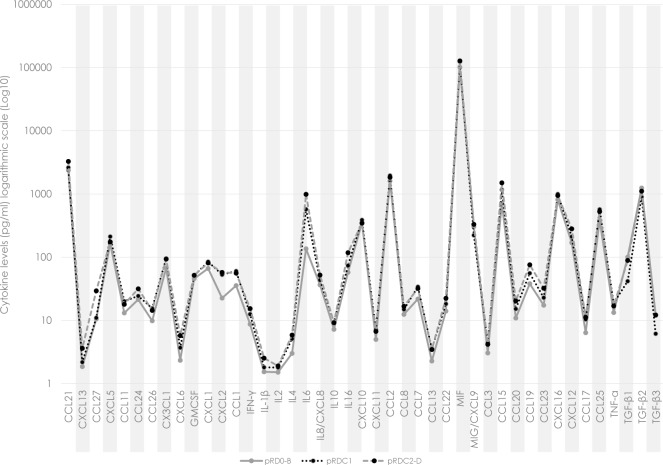
**Cytokine levels in the vitreous fluid regarding different PVR grades** Cytokine levels in the vitreous (VF) of eyes with primary retinal detachment without PVR or PVR grades A or B (pRD0-B; *n* = **81**) with PVR grade C1 (pRDC1; *n* = **27**) and grades C2-D (pRDC2-D; *n* = **65**).

Interestingly, comparing the vitreous levels of eyes with PVR C1 and PVR C2-D, no significant difference in chemo- and cytokine expression was found (Tables 4–6, Fig 3).

## Discussion

The comparison of cytokine profiles in different stages of proliferative vitreoretinopathy (PVR) in primary retinal detachment (pRD) identified the chemokine (C-C motif) ligand 19 (CCL19) as specifically upregulated in early PVR (C1). In more advanced stages of PVR (C2-D), 11 additional cytokines (CCL27, CXCL6, IL4, IL16, CXCL10, CCL8, CCL22, MIG/CXCL9, CCL15, CCL 23 and CXCL12) exhibited higher levels in the vitreous fluid compared to vitreous from eyes with pRD, but without PVR. When we compared the cytokine profiles in the pRD group with the most instable retinal situation that received silicone oil (group 3) to that of the group that received gas filling (group 2), CCL23 was the only cytokine elevated in the VF. Since 86.4% of group 3 showed severe PVR (C2-D) this cytokine might qualify as a marker for advanced PVR. This would be supported by the fact that CCL23 is induced by IL-4 and has chemotactic characteristics [25].

We found CCL19 to be the only cytokine upregulated in RD and PVR C1 compared to pRD with PVR grade B or less. CCl19 specifically binds to the chemokine receptor CCR7 which stimulates dendritic cell maturation [26]. Together with CCL21, CCL19 specifically binds to CCR7 and is constitutively expressed to control cell movement during homeostasis. CCR7 and CCL19 are also known to play a role in tissue repair and wound healing [27,28].

Samples of vitreous fluid from eyes with idiopathic epiretinal membranes and/or idiopathic macular holes have served in many studies as controls for cytokine analyses in various ocular pathologies [7,29,30]. Due to fibroblast activity in epiretinal membranes and an elevated inflammatory cytokine profile in the vitreous, we decided to use only patients with macular hole pathology without concomitant epiretinal membranes as a control group [31,32].

When compared to macular holes, the concentrations of almost all tested chemo- and cytokines were elevated in the vitreous of all pRD groups, irrespective of the presence or severity of PVR. This suggest that comparisons using only a single or very few cytokines may be misleading. TGF-beta 1- and 2 were the sole cytokines that were not significantly increased. Such unspecific upregulation of many cytokines may result from a damage to the retinovascular barrier following retinal detachment. Therefore, the cytokine milieu changes may represent a timely response to the tissue trauma which cannot be attributed to one single biological process [16,33,34,35]. The significant upregulation of pro-fibrotic and pro-inflammatory cytokines in eyes with RD compared to MH found in our study is in good agreement with previous studies that have found similar upregulations compared to eyes with either macular hole, epiretinal membrane or retinal vein occlusion, notably for cytokines as IL-6 and IL-8 [1,16,19,20,36,37], MCP1, MIP-1beta and IP10 [36,38], and in RD with PVR for IL-6, IL-8, IL-10, TNF, INF-gamma, CCL2, CCL3, CCL4, CCL5, CCL11, CCL17, CCL18, CCL19, CXCL9, CXCL19, G-CSF and FGF [2,12,39].

Our intention is a better understanding of the impact of local cytokine environmental changes in the progression of PVR. With the upregulation of 12 out of 43 chemo- and cytokines our findings do not indicate a very targeted or specific local response. This unspecific change in the intraocular environment during the progression from pRD to severe PVR, namely PVR grades C2-D may well explain why many therapeutic attempts to date remained unsuccessful [40,41].

Having identified CCL19 as a possible marker for the early detection of PVR, plus the subsequent upregulation of 11 additional chemo- and cytokines as PVR severity increases, seems to contradict the commonly held notion of this process being primarily unspecific. This raises the hope that we may at some point be able to specifically target the tissue response that results late in the progression of PVR. Currently, it is conceivable that CCL19 could serve as a diagnostic marker for the risk of PVR progression.

The main strength of this study is its well-designed selection process with sufficiently large numbers in each group. While the data is consistent, precise, and reliable, the storage conditions must be regarded as a weak point of this study. Samples were not immediately frozen due to the fact that our operation room is not close to the lab. This may principally allow a partial degradation of distinct peptides, chemo- and cytokines, so that we cannot exclude a minor difference in the absolute cytokine concentrations between sampling and storing. Nevertheless, this process was the same for all samples from all groups, and therefore cannot explain any of the inter-group differences we observed. Moreover, the cytokine concentrations in the aqueous humour and vitreous fluid reported here and in our previous studies [31,42,43] are well in line with the concentrations in the ocular fluids of published data from independent groups [44,45]. One further limitation of this study is the difference in age and gender between the groups. Both are known to play a role in the immune response [46,47,48,49,50,51]. Though their impact is unlikely to account for the differences found here, we have to assume that differences in age and gender might have added to the results.

In conclusion, we could identify substantial (but not targeted) changes in the cytokine profiles in pRD. This lends further support to the importance of unspecific cell activation processes over the course of the disease. However, the cellular source responsible for inducing the increased cytokine concentrations remains to be identified.

## Supporting information

S1 Dataset (XLSX)
